# Sospecha de hipersensibilidad probablemente relacionada con el uso de morfina: reporte de caso

**DOI:** 10.15446/rsap.V25n2.103211

**Published:** 2023-03-01

**Authors:** Roxana De las Salas, Gregorio Díaz-Morales, Juana Borja-González, Kevin A. Orta-Visbal, Laura Alvarado-Guerra, Donaldo E De la Hoz Santander

**Affiliations:** 1 RD: Enf. Universidad del Norte. M.Sc. Ciencias-Farmacología. Ph.D. Ciencias Farmacéuticas. Profesora Asistente, Departamento de Enfermería, Universidad del Norte. Barranquilla, Colombia. rdelassalas@uninorte.edu.co Universidad del Norte Universidad del Norte Departamento de Enfermería Barranquilla Colombia rdelassalas@uninorte.edu.co; 2 GD: QF. M.Sc. Toxicología. Docente Catedrático, Departamento de Enfermería, Universidad del Norte. Barranquilla, Colombia. gdiaz@uninorte.edu.co Universidad del Norte Departamento de Enfermería Universidad del Norte Barranquilla Colombia gdiaz@uninorte.edu.co; 3 JB: Enf. M.Sc. Enfermería. Ph.D. Educación. Profesora Asistente, Departamento de Enfermería, Universidad del Norte. Barranquilla, Colombia. gjuana@uninorte.edu.co Universidad del Norte Departamento de Enfermería Universidad del Norte Barranquilla Colombia gjuana@uninorte.edu.co; 4 KO: Enf. Esp. Cuidado Crítico del Adulto. M.Sc. Enfermería. Profesor instructor, Departamento de Enfermería, Universidad del Norte. Barranquilla, Colombia. korta@uninorte.edu.co Universidad del Norte Departamento de Enfermería Universidad del Norte Barranquilla Colombia korta@uninorte.edu.co; 5 LA: QF. Servicio Farmacéutico, Hospital Universidad del Norte. Barranquilla, Colombia. qfasistencial@uninorte.edu.co Hospital Universidad del Norte Servicio Farmacéutico Hospital Universidad del Norte Barranquilla Colombia; 6 DDH: QF. Esp. Farmacia Clínica. Jefe de Farmacia, Hospital Universidad del Norte. Docente Catedrático, Universidad del Atlántico. Barranquilla, Colombia. santanderd@uninorte.edu.co Universidad del Atlántico Universidad del Atlántico Barranquilla Colombia santanderd@uninorte.edu.co

**Keywords:** Morfina, efectos colaterales y reacciones adversas relacionados con medicamentos, hipersensibilidad, farmacovigilancia *(fuente: DeCS, BIREME)*, Morphine, drug-related side effects and adverse reactions, hypersensitivity, pharmacovigilance *(source*, *MeSH, NLM)*

## Abstract

Se presenta el caso de un paciente masculino de 46 años. Este ingresó al servicio de urgencias con un cuadro clínico de una hora de evolución caracterizado por dolor torácico opresivo de intensidad 10/10 según la escala análoga del dolor. Después de la valoración y frente a los hallazgos electrocardiográficos y de laboratorio, se diagnosticó al paciente con infarto agudo de miocardio con elevación del ST, además, se sospechó de disección aórtica y de una emergencia hipertensiva a órgano blanco corazón debido al intenso dolor precordial y las cifras tensionales de 230/120 mmHg. Dentro del esquema terapéutico, se indicaron 3 mg de morfina intravenosa diluida en 10 ml de solución salina 0,9%. Después de la administración, el paciente presentó sospecha de hipersensibilidad. Por ende, se realizó una evaluación de sospecha de evento adverso utilizando el algoritmo de Naranjo y se estableció que los efectos de morfina eran plausibles (categoría probable).

## Abstract (en)

The case of a 46-year-old male patient is presented. He was admitted to the emergency department with a clinical picture of oppressive chest pain of 10/10 intensity on the pain analog scale, which had been evolving for one hour. After evaluation and based on electrocardiographic and laboratory findings, the patient was diagnosed with acute myocardial infarction with st elevation. Additionally, aortic dissection and hypertensive emergency with end-organ damage to the heart were suspected due to intense precordial pain and blood pressure readings of 230/120 mmHg. As part of the therapeutic approach, 3 mg of intravenous morphine diluted in 10 ml of 0.9 % saline solution were administered. Following administration, the patient exhibited suspected hypersensitivity. Therefore, a suspected adverse event assessment was performed using the Naranjo algorithm, and it was established that the effects of morphine were plausible (category probable).

La morfina es un analgésico opioide que actúa como agonista de los receptores opioides en el sistema nervioso central (SNC), principalmente en los receptores kappa. Estos receptores están relacionados con la analgesia espinal, la miosis y la sedación, mientras que los receptores mu son los mediadores de la analgesia supraespinal, la depresión respiratoria y la euforia. Además, la morfina ejerce un efecto directo sobre el plexo nervioso de la pared intestinal 1.

Las reacciones adversas por morfina muy frecuentes (aquellas que se producen en >i/io pacientes) que pueden aparecer a dosis terapéuticas, son las gastrointestinales (náuseas y estreñimiento). Dentro de las frecuentes (≥1/100, <1/100 pacientes) se encuentran las gastrointestinales (vómitos) y los trastornos del sistema nervioso (somnolencia). Las reacciones adversas de frecuencia no conocida que pueden aparecer (y no pueden estimarse a partir de los datos disponibles), son los trastornos del sistema inmunológico (reacciones anafilactoides). Aunque se consideran estas últimas entre poco frecuentes (≥1/1.000, <1/100 pacientes) y raras (≥1.10.000, <1/1.000 pacientes), son las que más llaman la atención dada la rareza y la gravedad con que se producen 1.

Las reacciones de hipersensibilidad a fármacos (RHF) se refieren a síntomas o signos objetivamente reproducibles iniciados por la exposición a una formulación farmacéutica (incluidos los fármacos activos y los excipientes) a una dosis normalmente tolerada por personas no hipersensibles. Son un tipo de efecto adverso a medicamento impredecible e incluyen reacciones inducidas por células inmunitarias o inflamatorias, así como otros mecanismos no inmunológicos. Las RHF pertenecen a las reacciones adversas a medicamentos de tipo B que la Organización Mundial de la Salud (OMS) define como la respuesta independiente de la dosis, impredecible, nociva y no intencionada a un fármaco tomado en una dosis normalmente utilizada en humanos. Asimismo, las reacciones de hipersensibilidad a fármacos se clasifican clínicamente como reacciones inmediatas (que aparecen dentro de i hora después de la ingesta del fármaco) y no inmediatas (que aparecen > i hora después de la ingesta del fármaco) 2.

Por otro lado, de acuerdo con la guía de tratamiento del infarto agudo de miocardio con elevación del segmento ST de la Sociedad Europea de Cardiología, el alivio del dolor es de suma importancia, dado que la activación simpática derivada de este se relaciona con vasoconstricción y aumento de la carga de trabajo del corazón 3.

Dada la baja incidencia que tienen los eventos de hipersensibilidad relacionados con el uso de morfina y el interés que los efectos adversos a medicamentos tienen en la salud pública y la comunidad académica y asistencial, se analiza la presentación de un caso probable de hipersensibilidad relacionado con la administración de morfina intravenosa y se establece la metodología de análisis de caso que permite alertar sobre su ocurrencia y potencial gravedad.

## PRESENTACIÓN DEL CASO

A las 03:38am ingresó un hombre de 46 años consciente y orientado a la institución con un cuadro clínico de una hora de evolución, caracterizado por dolor torácico opresivo de intensidad 10/10 según escala análoga del dolor. El dolor se irradiaba al tórax y afirmó tener antecedentes de hipertensión arterial en tratamiento con losartán 50 mg vía oral cada 12 horas. Negó ser alérgico a medicamentos, pero no se obtuvo información sobre el cumplimiento y la adherencia al tratamiento.

Después, un médico general lo evaluó y lo trasladó al área de reanimación. Se observó palidez generalizada, criodiaforesis y cabeza normocefálica. Además, se encontraron pupilas isocóricas normo-reactivas a la luz, fosas nasales permeables que toleraban el oxígeno ambiente, mucosa oral seca, cuello móvil y sin masas, tórax simétrico normo-expandible, abdomen globoso, pero no doloroso a la palpación, genitales externos normoconfigurados cubiertos, extremidades simétricas y móviles, y piel íntegra. A continuación, se inició la monitorización de los signos vitales.

Se diagnosticó al paciente con infarto agudo de miocardio con elevación del ST, además se sospechó de disección aórtica y de una emergencia hipertensiva a órgano blanco corazón, debido al intenso dolor precordial y las cifras tensionales de 230/120 mmHg.

Acto seguido, se intentó canalizar una vía periférica con un catéter N°i8 en el dorso de la mano del miembro superior derecho, pero el intento fue fallido. En su lugar, se instaló un conector en Y libre de agujas, se colocaron 1000 ml de líquidos endovenosos de SSN 0,9% y se fijó sin complicaciones. Luego, se llevaron a cabo los paraclínicos que se reportan en la Tabla 1.

**Tabla 1:** Resultados de paraclínicos en sangre

Nombre de paraclinico	Resultado (Valor de referencia)
Tiempo de protrombina	10 segundos (10,6 segundos)
Tiempo de tromboplastina	23,4 segundos (25,7 segundos)
INR	0,93
Sodio en sangre	151,2 meq/l (135 - 145)
Potasio en sangre	3,84 meq/l (3,5 - 5,0)
Troponina	0,03 ng/ml (0,0-0,06)
Glicemia	117 mg/dl (60 - 110 mg/dl)
BUN [Nitrógeno Uréico]	11,41 mg/dl (5 - 25)
UREA	24,42 mg/dl (10 - 50)
Creatinina en sangre	1,61 mg/dl (0,5 -1,30)

Se prescribió e inició tratamiento con nitroglicerina 5o mg a razón de 10ml/h por bomba de infusión. Seguidamente, se administró morfina 3 mg en 10 ml de solución salina normal (SSN) 0.9%. Posterior a la administración de morfina, el paciente presentó inmediatamente ronchas o habones eritematosas en el antebrazo derecho, en el sitio de venopunción, y prurito local. Por este motivo, se le prescribió hidroxicina 100 miligramos intramuscular, dosis única, la cual mejoró el cuadro cutáneo. Después, el paciente presentó una hipotensión sostenida, criodiaforesis y palidez generalizada.

Se permeabilizó la vía periférica con SSN 0,9% y se inició la sedación con fentanilo 100 mcg, midazolam 5 mg y vecuronio 5 mg intravenoso. Se procedió a asegurar la vía aérea con un tubo 8.0 c/b sin complicaciones y se instaló un filtro hidroscópico y sujetador de tubo de circuito ventilatorio, que se conectó a un ventilador mecánico bajo parámetros establecidos. Se puso sonda orogástrica # 18 y se observó la salida de jugo gástrico sin complicaciones.

Se inició infusión de norepinefrina 8 mg en 100 cm^3^ de SSN 0,9% a razón de 0,2 mcg/kg/min por bomba de infusión. Se continuó con la administración de líquidos endovenosos con Hartman 500 ml a 120 ml/h por bomba de infusión. El paciente entró en paro cardio-respiratorio, se activó el código azul y se iniciaron maniobras de reanimación básicas y avanzadas. Se administró tratamiento con adrenalina a intervalos de 3 minutos, para un total de 6. Después de 16 minutos con actividad eléctrica y sin pulso (que progresó a asistolia) el paciente falleció.

### Evaluación

Teniendo en cuenta que el paciente presentó una cascada de eventos, se suministra la evaluación de sospecha de hipersensibilidad asociado a la administración de morfina. En la Tabla 2, se muestra la evaluación del caso con el algoritmo de Naranjo, con un puntaje de 6, lo cual indica una probable asociación del efecto adverso con el uso de morfina.

**Tabla 2:** Análisis con el Algoritmo de Naranjo

Preguntas	Sí	No	No se sabe	Puntos
1. ¿Existen notificaciones concluyentes sobre esta reacción?	1	0	0	1
2. ¿Se produjo la RA después de administrar el fármaco sospechoso?	2	-1	0	2
3. ¿Mejoró la RA tras suspender la administración del fármaco o tras administrar un antagonista específico?	1	0	0 (x)	0
4. ¿Reapareció la RA tras la re-administración del fármaco?	2	-1	0	0
5. ¿Existen causas alternativas (diferentes del fármaco) que podrían haber causado la reacción por sí misma?	-1	2	0	2
6. ¿Reapareció la RA tras administrar placebo?	-1	1	0	0
7. ¿Se detectó el fármaco en la sangre (o en otros fluidos) en concentraciones tóxicas?	1	0 (x)	0	0
8. ¿Fue la reacción más severa al aumentar la dosis o menos severa al disminuirla?	1	0	0 (x)	0
9. ¿Tuvo el paciente alguna reacción similar causada por el mismo fármaco u otro semejante en cualquier exposición anterior?	1	0 (x)	0	0
10. ¿Se confirmó el acontecimiento adverso por cualquier tipo de evidencia objetiva?	1	0	0	1
Puntuación total	6			
Las categorías correspondientes a la puntuación total son las siguientes: La reacción adversa a medicamentos es: segura: ≥ 9; probable: 5-8; posible: 1-4; improbable: 0.				

Teniendo en cuenta la temporalidad del evento y los aspectos farmacológicos del medicamento sospechoso, se considera plausible la asociación entre el uso de morfina y el desarrollo de cuadros cutáneos y cardiovasculares presentados. Se ha documentado que aproximadamente un 3% de los pacientes que reciben morfina presentan reacciones adversas a nivel cardiovascular 1.

La evaluación de la mejoría del evento tras la retirada del producto y/o la administración de antídotos, no se puede determinar teniendo en cuenta el abordaje instaurado en el contexto clínico del paciente. Para el caso de los opiáceos, el antídoto de elección es naloxona. Si bien no fue posible determinar concentraciones séricas de morfina en rangos de toxicidad, al tratarse de un antagonista competitivo de los receptores de opioides, revierte la unión de morfina a los receptores, pero no sobre los mecanismos inmunológicos y mediadores de las reacciones de hipersensibilidad.

Se establece, además, que existen manifestaciones clínicas del evento que pueden estar superpuestas con la reacción adversa al medicamento, el cual fue aplicado en las dosis adecuadas y no hubo reexposición a este, dado que fue prescrito en dosis única. En lo concerniente al paciente, este no manifiesta ningún tipo de antecedente de alergias a medicamentos u otras sustancias que pudiesen contribuir al cuadro clínico. De igual manera, dentro del tratamiento farmacológico instaurado, no se detectan interacciones medicamentosas potenciales que estén contribuyendo a la aparición de este evento.

## DISCUSIÓN

De acuerdo con la guía de tratamiento del infarto agudo de miocardio con elevación del segmento ST de la Sociedad Europea de Cardiología, la administración intravenosa de analgésicos opioides como morfina tiene un grado de recomendación I y nivel de evidencia C 3. La ficha técnica establece la administración intravenosa directa de 2,5 - 15 mg de morfina diluidos en 4-5 ml de agua estéril para inyección o con solución de cloruro de sodio al 0,9%, para administrar lentamente durante 4-5 minutos 1. Lo anterior, sugiere una adecuada indicación y forma de administración de la morfina en el caso presentado, sin embargo, el paciente desarrolla una probable reacción de hipersensibilidad al fármaco. Asimismo, se resaltan dos formas de presentación de los efectos adversos a morfina, caracterizados inicialmente por las manifestaciones cutáneas, probablemente por la administración de morfina endovenosa, que evolucionan a eventos cardiovasculares y muerte.

Es importante revisar los efectos posibles de la reacción anafiláctica como agravantes de la situación coronaria, teniendo en cuenta que, con la descarga sistémica de histamina durante la respuesta inmune, se aumenta la permeabilidad de los capilares sanguíneos con la consecuente extravasación de líquido que se traduce en disminución de la precarga, con posterior deterioro del gasto cardíaco.

Según la base de datos VigiAccess, los efectos adversos en el sitio de administración y desórdenes de tipo inmune cobran un gran volumen de reportes de seguridad relacionados con morfina, sin embargo, su incidencia esperada es baja. Esto puede deberse a que los efectos de hiper-sensibilidad son más fáciles de reconocer y que dado el especial interés que hay en la identificación y seguimiento, se puede favorecer su reporte en los sistemas de farmacovigilancia 4.

A la valoración del algoritmo de Naranjo, se observó una puntuación para categoría de probable. No fue posible obtener la categoría de segura debido a que algunas preguntas del algoritmo como la readministración del medicamento y la valoración de cambios después de la administración de la hidroxicina no se pudo establecer dado el desenlace que tuvo el cuadro clínico del paciente. Tampoco es posible establecer si los efectos pueden relacionarse a los excipientes de la formulación medicamentosa, sin embargo, no se puede descartar esta posibilidad 5.

Desde el punto de vista clínico, las RHF se pueden clasificar inmediatas si ocurren dentro de 1 hora después de la última administración del medicamento y no inmediatas si se presentan a partir de 1 hora después de la administración inicial del medicamento 2. Las RHF alérgicas inmediatas se desarrollan como resultado de la producción de IgE por los linfocitos B específicos de antígeno luego de la sensibilización. Los anticuerpos IgE se unen a sus receptores de alta afinidad en la superficie de los mastocitos y basófilos, creando un sitio de unión multivalente para el antígeno del fármaco. Después de la exposición, el antígeno (complejo hapteno-proteína) se entrecruza, mientas que la IgE continúa unida y estimula la liberación de mediadores preformados (histamina, triptasa, algunas citoquinas como TNF-a) y la producción de nuevos mediadores (leucotrienos, prostaglandinas, cininas, otras citoquinas). A su vez, los mediadores preformados estimulan una respuesta en cuestión de minutos, mientras que el componente inflamatorio de citocinas se desarrolla después de varias horas, el tiempo necesario para la síntesis de proteínas y el reclutamiento de células inmunitarias. Bajo este elemento, la anafilaxia por morfina produce una RHF alérgica inmediata, mientras que la mayoría de las RHF alérgicas no inmediatas (tardías) están mediadas por las acciones de los linfocitos T 6.

Dada la presentación clínica del evento, se considera probable la reacción de hipersensibilidad a morfina que permite alertar sobre los efectos de hipersensibilidad. Se ha demostrado la reacción de hipersensibilidad inducida por analgésicos opioides en humanos. En consecuencia, se resalta la importancia de los protocolos de medicamentos para asegurar el uso adecuado de morfina desde su indicación hasta su administración ♣
